# Two Different Methods for Numerical Solution of the Modified Burgers' Equation

**DOI:** 10.1155/2014/780269

**Published:** 2014-04-03

**Authors:** Seydi Battal Gazi Karakoç, Ali Başhan, Turabi Geyikli

**Affiliations:** ^1^Department of Mathematics, Faculty of Science and Art, Nevsehir Haci Bektas Veli University, 50300 Nevsehir, Turkey; ^2^Department of Mathematics, Faculty of Science and Art, Inonu University, 44280 Malatya, Turkey

## Abstract

A numerical solution of the modified Burgers' equation (MBE) is obtained by using quartic B-spline subdomain finite element method (SFEM) over which the nonlinear term is locally linearized and using quartic B-spline differential quadrature (QBDQM) method. The accuracy and efficiency of the methods are discussed by computing *L*_2_ and *L*_*∞*_ error norms. Comparisons are made with those of some earlier papers. The obtained numerical results show that the methods are effective numerical schemes to solve the MBE. A linear stability analysis, based on the von Neumann scheme, shows the SFEM is unconditionally stable. A rate of convergence analysis is also given for the DQM.

## 1. Introduction

The one-dimensional Burgers' equation first suggested by Bateman [1] and later treated by Burgers' [2] has the form

(1) $$\begin{array}{c} U_{t}+UU_{x}-vU_{xx}=0, \end{array}$$

where *v* is a positive parameter and the subscripts *x* and *t* denote space and time derivatives, respectively. Burgers' model of turbulence is very important in fluid dynamics model and study of this model and the theory of shock waves has been considered by many authors for both conceptual understanding of a class of physical flows and for testing various numerical methods [3]. Relationship between (1) and both turbulence theory and shock wave theory was presented by Cole [4]. He also obtained an exact solution of the equation. Analytical solutions of the equation were found for restricted values of *v* which represent the kinematics viscosity of the fluid motion. So the numerical solution of Burgers' equation has been subject of many papers. Various numerical methods have been studied based on finite difference [5, 6], Runge-Kutta-Chebyshev method [7, 8], group-theoretic methods [9], and finite element methods including Galerkin, Petrov-Galerkin, least squares, and collocation [10–13]. The modified Burgers' equation (MBE) which we discuss in this paper is based upon Burgers' equation (BE) of the form

(2) $$\begin{array}{c} U_{t}+U^{2}U_{x}-vU_{xx}=0. \end{array}$$

The equation has the strong nonlinear aspects and has been used in many practical transport problems, for instance, nonlinear waves in a medium with low-frequency pumping or absorption, turbulence transport, wave processes in thermoelastic medium, transport and dispersion of pollutants in rivers and sediment transport, and ion reflection at quasi-perpendicular shocks. Recently, some numerical studies of the equation have been presented: Ramadan and El-Danaf [14] obtained the numerical solutions of the MBE using quintic B-spline collocation finite element method. A special lattice Boltzmann model is developed by Duan et al. [15]. Dağ et al. [16] have developed a Galerkin finite element solution of the equation using quintic B-splines and time-split technique. A solution based on sextic B-spline collocation method is proposed by Irk [17]. Roshan and Bhamra [18] applied a Petrov-Galerkin method using a linear hat function as the trial function and a cubic B-spline function as the test function. A discontinuous Galerkin method is presented by Zhang et al. [19]. Bratsos [20] has used a finite difference scheme based on fourth-order rational approximants to the matrix-exponential term in a two-time level recurrence relation for calculating the numerical solution of the equation.

Recently, DQM has become a very efficient and effective method to obtain the numerical solutions of various types of partial differential equations. In 1972, Bellman et al. [21] first introduced differential quadrature method (DQM) for solving partial differential equations. The main idea behind the method is to find out the weighting coefficients of the functional values at nodal points by using base functions of which derivatives are already known at the same nodal points over the entire region. Various researchers have developed different types of DQMs by utilizing various test functions; Bellman et al. [22] have used Legendre polynomials and spline functions in order to get weighting coefficients. Quan and Chang [23, 24] have presented an explicit formulation for determining the weighting coefficients using Lagrange interpolation polynomials. Zhong [25], Guo and Zhong [26], and Zhong and Lan [27] have introduced another efficient DQM as spline based DQM and applied it to different problems. Shu and Wu [28] have considered some of the implicit formulations of weighting coefficients with the help of radial basis functions. Nonlinear Burgers' equation is solved using polynomial based differential quadrature method by Korkmaz and Dağ [29]. The DQM has many advantages over the classical techniques; mainly, it prevents linearization and perturbation in order to find better solutions of given nonlinear equations. Since QBDQM do not need transforming for solving the equation, the method has been preferred.

In the present work, we have applied a subdomain finite element method and a quartic B-spline differential quadrature method to the MBE. To show the performance and accuracy of the methods and make comparisons of numerical solutions, we have taken different values of *v*.

## 2. Numerical Methods

To implement the numerical schemes, the interval [*a*, *b*] is splitted up into uniformly sized intervals by the nodes *x*_*m*_, *m* = 1,2,…, *N*, such that *a* = *x*_0_ < *x*_1_ ⋯ <*x*_*N*_ = *b*, where *h* = (*x*_*m*+1_ − *x*_*m*_).

### 2.1. Subdomain Finite Element Method (SFEM)

We will consider (2) with the boundary conditions chosen from

(3) $$\begin{array}{c} U\left(a,t\right)=\beta_{1},\;\;U\left(b,t\right)=\beta_{2},\\ U_{x}\left(a,t\right)=0,\;\;U_{x}\left(b,t\right)=0,\\ U_{xx}\left(a,t\right)=0,\;\;U_{xx}\left(b,t\right)=0,\;\;t>0, \end{array}$$

with the initial condition

(4) $$\begin{array}{c} U\left(x,0\right)=f\left(x\right),\;a\leq x\leq b, \end{array}$$

where *β*_1_ and *β*_2_ are constants. The quartic B-splines *ϕ*_*m*_(*x*) (*m* = −2(1)  *N* + 1) at the knots *x*_*m*_ which form a basis over the interval [*a*, *b*] are defined by the relationships [30]

(5) ϕm(x)=1h4{(x−xm−2)4,x∈[xm−2,xm−1],(x−xm−2)4−5(x−xm−1)4,x∈[xm−1,xm],(x−xm−2)4−5(x−xm−1)4+10(x−xm)4,x∈[xm,xm+1],(xm+3−x)4−5(xm+2−x)4,x∈[xm+1,xm+2],(xm+3−x)4,x∈[xm+2,xm+3],0,otherwise.

Our numerical treatment for solving the MBE using the subdomain finite element method with quartic B-splines is to find a global approximation *U*_*N*_(*x*, *t*) to the exact solution *U*(*x*, *t*) that can be expressed in the following form:

(6) $$\begin{array}{c} U_{N}\left(x,t\right)=\overset{N+1}{\underset{j=-2}{\sum }}\delta_{j}\left(t\right)\phi_{j}\left(x\right), \end{array}$$

where *δ*_*j*_ are time-dependent parameters to be determined from both boundary and weighted residual conditions. The nodal values *U*_*m*_, *U*_*m*_′, *U*_*m*_′′, and *U*_*m*_′′′ at the knots *x*_*m*_ can be obtained from (5) and (6) in the following form:

(7) $$\begin{array}{c} U_{m}=U\left(x_{m}\right)=\delta_{m-2}+11\delta_{m-1}+11\delta_{m}+\delta_{m+1},\\ U_{m}^{\prime }=U^{\prime }\left(x_{m}\right)=\frac{4}{h}\left(-\delta_{m-2}-3\delta_{m-1}+3\delta_{m}+\delta_{m+1}\right),\\ U_{m}^{\prime \prime }=U^{\prime \prime }\left(x_{m}\right)=\frac{12}{h^{2}}\left(\delta_{m-2}-\delta_{m-1}-\delta_{m}+\delta_{m+1}\right),\\ U_{m}^{\prime \prime \prime }=U^{\prime \prime \prime }\left(x_{m}\right)=\frac{24}{h^{3}}\left(-\delta_{m-2}+3\delta_{m-1}-3\delta_{m}+\delta_{m+1}\right). \end{array}$$

For each element, using the local coordinate transformation

(8) $$\begin{array}{c} h\xi =x-x_{m},\;0\leq \xi \leq 1, \end{array}$$

a typical finite interval [*x*_*m*_, *x*_*m*+1_] is mapped into the interval [0,1]. Therefore, the quartic B-spline shape functions over the element [0,1] can be defined as

(9) $$\begin{array}{c} \phi^{e}=\{\begin{array}{cc} \phi_{m-2}=1-4\xi +6\xi^{2}-4\xi^{3}+\xi^{4}, & \\ \phi_{m-1}=11-12\xi -6\xi^{2}+12\xi^{3}-\xi^{4}, & \\ \phi_{m}=11+12\xi -6\xi^{2}-12\xi^{3}+\xi^{4}, & \\ \phi_{m+1}=1+4\xi +6\xi^{2}+4\xi^{3}-\xi^{4}, & \\ \phi_{m+2}=\xi^{4}. & \end{array} \end{array}$$

All other splines, apart from *ϕ*_*m*−2_(*x*), *ϕ*_*m*−1_(*x*), *ϕ*_*m*_(*x*), *ϕ*_*m*+1_(*x*), and *ϕ*_*m*+2_(*x*), are zero over the element [0,1]. So, the approximation equation (6) over this element can be written in terms of basis functions given in (9) as

(10) $$\begin{array}{c} U_{N}\left(\xi ,t\right)=\overset{m+2}{\underset{j=m-2}{\sum }}\delta_{j}\left(t\right)\phi_{j}\left(\xi \right), \end{array}$$

where *δ*_*m*−2_, *δ*_*m*−1_, *δ*_*m*_, *δ*_*m*+1_, and *δ*_*m*+2_ act as element parameters and B-splines *ϕ*_*m*−2_(*x*), *ϕ*_*m*−1_, *ϕ*_*m*_, *ϕ*_*m*+1_, and *ϕ*_*m*+2_ as element shape functions. Applying the subdomain approach to (33) with the weight function

(11) $$\begin{array}{c} W_{m}\left(x\right)=\{\begin{array}{cc} 1, & x\in \left[x_{m},x_{m+1}\right],\\ 0, & \text{otherwise} \end{array} \end{array}$$

we obtain the weak form of (2)

(12) $$\begin{array}{c} \overset{x_{m+1}}{\underset{x_{m}}{\int }}1.\left(U_{t}+U^{2}U_{x}-vU_{xx}\right)dx=0. \end{array}$$

Using the transformation (8) into the weak form (12) and then integrating (12) term by term with some manipulation by parts result in

(13) h5(δ˙m−2+26δ˙m−1+66δ˙m+26δ˙m+1+δ˙m+2)  +Zm(−δm−2−10δm−1+10δm+1+δm+2)  −4vh(δm−2+2δm−1−6δm+2δm+1+δm+2)=0,

where the dot denotes differentiation with respect to *t*, and

(14) $$\begin{array}{c} Z_{m}={\left(\delta_{m-2}+11\delta_{m-1}+11\delta_{m}+\delta_{m+1}\right)}^{2}. \end{array}$$

In (13) using the Crank-Nicolson formula and its time derivative that is discretized by the forward difference approach, respectively,

(15) $$\begin{array}{c} \delta_{m}=\frac{\delta_{m}^{n}+\delta_{m}^{n+1}}{2},\;\;{\dot{\delta }}_{m}=\frac{\delta_{m}^{n+1}-\delta_{m}^{n}}{\Delta t} \end{array}$$

we obtain a recurrence relationship between the two time levels *n* and *n* + 1 relating two unknown parameters *δ*_*i*_^*n*+1^ and *δ*_*i*_^*n*^, for *i* = *m* − 2, *m* − 1,…, *m* + 2,

(16) αm1δm−2n+1+αm2δm−1n+1+αm3δmn+1+αm4δm+1n+1   +αm5δm+2n+1  =αm6δm−2n+αm7δm−1n+αm8δmn+αm9δm+1n   +αm10δm+2n,m=0,1,…,N−1,

where

(17) αm1=1−EZm−M,  αm2=26−10EZm−2M,αm3=66+6M,  αm4=26+10EZm−2M,αm5=1+EZm−M,  αm6=1+EZm+M,αm7=26+10EZm+2M,  αm8=66−6M,αm9=26−10EZm+2M,αm10=1−EZm+M,E=5Δt2h,M=20vΔt2h2.

Obviously, the system (16) consists of *N* equations in the *N* + 4 unknowns (*δ*_−2_, *δ*_−1_,…, *δ*_*N*+1_). To get a unique solution of the system, we need four additional constraints. These are obtained from the boundary conditions (3) and can be used to eliminate *δ*_−2_, *δ*_−1_, *δ*_*N*_, and *δ*_*N*+1_ from the system (16) which then becomes a matrix equation for the *N* unknowns *d* = (*δ*_0_, *δ*_1_,…, *δ*_*N*−1_) of the form

(18) $$\begin{array}{c} Ad^{n+1}=Bd^{n}. \end{array}$$

A lumped value of *Z*_*m*_ is obtained from (*U*_*m*_ + *U*_*m*+1_)^2^/4 as

(19) $$\begin{array}{c} Z_{m}=\frac{1}{4}{\left(\delta_{m-2}+12\delta_{m-1}+22\delta_{m}+12\delta_{m+1}+\delta_{m+2}\right)}^{2}. \end{array}$$

The resulting system can be solved with a variant of Thomas algorithm and we need an inner iteration (δ*)^*n*+1^ = *δ* ^*n*^ + (1/2)(*δ* ^*n*+1^ − *δ* ^*n*^) at each time step to cope with the nonlinear term *Z*_*m*_. A typical member of the matrix system (16) is rewritten in terms of the nodal parameters *δ*_*m*_^*n*^ as

(20) γ1δm−2n+1+γ2δm−1n+1+γ3δmn+1+γ4δm+1n+1+γ5δm+2n+1  =γ6δm−2n+γ7δm−1n+γ8δmn+γ9δm+1n+γ10δm+2n,

where

(21) γ1=α−β−λ,  γ2=26α−10β−2λ,γ3=66α+6λ,  γ4=26α+10β−2λ,γ5=α+β−λ,  γ6=α+β+λ,γ7=26α+10β+2λ,  γ8=66α−6λ,γ9=26α−10β+2λ,  γ10=α−β+λ,α=1, β=EZm, λ=M.

Before the solution process begins iteratively, the initial vector *δ* ^0^ = (*δ*_0_, *δ*_1_,…, *δ*_*N*−1_) must be determined by means of the following requirements:

(22) U′(a,0)=4h(−δ−20−3δ−10+3δ00+δ10)=0,U′′(a,0)=12h2(δ−20−δ−10−δ00+δ10)=0,U(xm,0)=δm−20+11δm−10+11δm0+δm+10=f(x),m=0,1,…,N−1,U′(b,0)=4h(−δN−20−3δN−10+3δN0+δN+10)=0,U′′(b,0)=12h2(δN−20−δN−10−δN0+δN+10)=0.

If we eliminate the parameters *δ*_−2_^0^, *δ*_−1_^0^, *δ*_*N*_^0^, and *δ*_*N*+1_^0^ from the system (16), we obtain *N* × *N* matrix system of the following form:

(23) $$\begin{array}{c} A\delta^{0}=B, \end{array}$$

where *A* is

(24) $$\begin{array}{c} A=\left[\begin{array}{cccccccc} 18 & 6 & & & & & & \\ 11.5 & 11.5 & 1 & & & & & \\ 1 & 11 & 11 & 1 & & & & \\ & & & & 1 & 11 & 11 & 1\\ & & & & & 2 & 14 & 8 \end{array}\right], \end{array}$$

*δ* ^0^ = [*δ*_0_^0^, *δ*_1_^0^,…, *δ*_*N*−1_^0^]^*T*^, and *B* = [*U*(*x*_0_, 0), *U*(*x*_1_, 0),…, *U*(*x*_*N*−1_, 0)]^*T*^. This system is solved by using a variant of Thomas algorithm.

### 2.2. Linear Stability Analysis

We have investigated stability of the scheme by using the von Neumann method. In order to apply the stability analysis, the MBE needs to be linearized by assuming that the quantity *U* in the nonlinear term *U* ^2^ *U*_*x*_ is locally constant. The growth factor of a typical Fourier mode is defined as

(25) $$\begin{array}{c} \delta_{j}^{n}=\xi^{n}e^{ijkh}, \end{array}$$

where *k* is mode number and *h* is the element size. Substituting (37) into the scheme (20), we have

(26) $$\begin{array}{c} g=\frac{A_{1}+ib}{A_{2}-ib}, \end{array}$$

where

(27) $$\begin{array}{c} A_{1}=\left(\alpha -\lambda \right)\cos\left(2kh\right)+\left(26\alpha -2\lambda \right)\cos\left(kh\right)+66+6\lambda ,\\ A_{2}=\left(\alpha +\lambda \right)\cos\left(2kh\right)+\left(26\alpha +2\lambda \right)\cos\left(kh\right)+66-6\lambda ,\\ b=\sin\left(2kh\right)+10\sin\left(kh\right). \end{array}$$

We can see that *A*_1_^2^ < *A*_2_^2^ and taking the modulus of (38) gives |*g* | ≤1, so we find that the scheme (20) is unconditionally stable.

### 2.3. Quartic B-Spline Differential Quadrature Method (QBDQM)

DQM can be defined as an approximation to a derivative of a given function by using the linear summation of its values at specific discrete nodal points over the solution domain of a problem. Provided that any given function *U*(*x*) is enough smooth over the solution domain, its derivatives with respect to *x* at a nodal point *x*_*i*_ can be approximated by a linear summation of all the functional values in the solution domain, namely,

(28) Ux(r)(xi)=dU(r)dx(r)|xi=∑j=1Nwij(r)U(xj),i=1,2,…,N, r=1,2,…,N−1,

where *r* denotes the order of the derivative, *w*_*ij*_^(*r*)^ represent the weighting coefficients of the *r*th order derivative approximation, and *N* denotes the number of nodal points in the solution domain. Here, the index *j* represents the fact that *w*_*ij*_^(*r*)^ is the corresponding weighting coefficient of the functional value *U*(*x*_*j*_). We need first- and second-order derivative of the function *U*(*x*). So, we will find value of (28) for the *r* = 1,2. If we consider (28), then it is seen that the fundamental process for approximating the derivatives of any given function through DQM is to find out the corresponding weighting coefficients *w*_*ij*_^(*r*)^. The main idea of the DQM approximation is to find out the corresponding weighting coefficients *w*_*ij*_^(*r*)^ by means of a set of base functions spanning the problem domain. While determining the corresponding weighting coefficients different basis may be used. Using the quartic B-splines as test functions in the fundamental DQM equation (28) leads to the equation

(29) d(r)Qm(xi)dx(r)=∑j=m−1m+2wi,j(r)Qm(xj),m=−1,0,…,N+2, i=1,2,…,N.

### 2.4. First-Order Derivative Approximation

When DQM methodology is applied, the fundamental equality for determining the corresponding weighting coefficients of the first-order derivative approximation is obtained as Korkmaz used [31]

(30) dQm(xi)dx=∑j=m−1m+2wi,j(1)Qm(xj),m=−1,0,…,N+1, i=1,2,…,N.

In this process, the initial step for finding out the corresponding weighting coefficients *w*_*i*,*j*_^(1)^, *j* = −2, −1,…, *N* + 3, of the first nodal point *x*_1_ is to apply the test functions *Q*_*m*_,  *m* = −1,0,…, *N* + 1, at the nodal point *x*_1_. After all the *Q*_*m*_ test functions are applied, we get the following system of algebraic equation system:

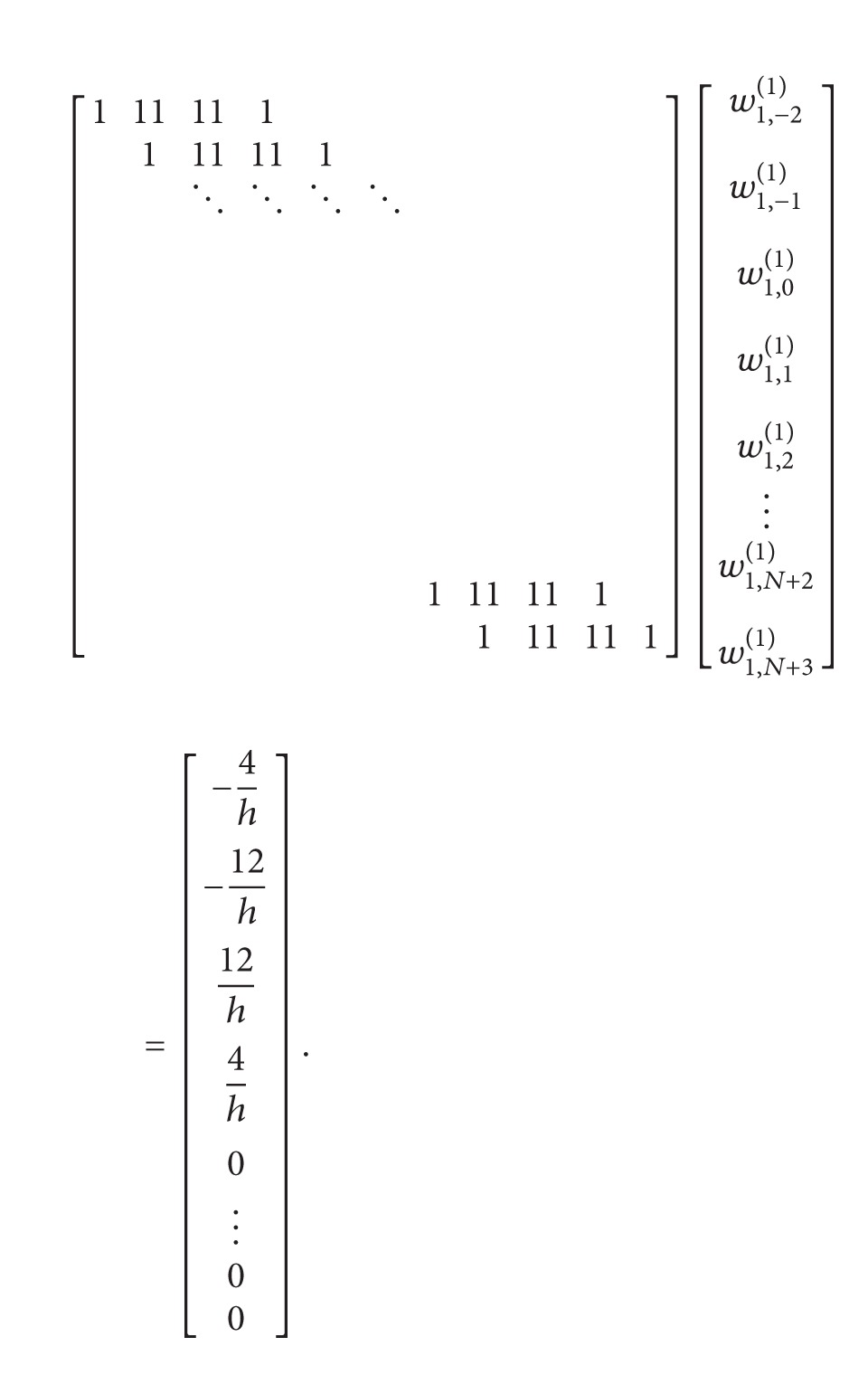
(31)


The weighting coefficients *w*_1,*j*_^(1)^ related to the first grid point are determined by solving the system (31). This system consists of *N* + 6 unknowns and *N* + 3 equations. To have a unique solution, it is required to add three additional equations to the system. These are

(32) $$\begin{array}{c} \frac{d^{\left(2\right)}Q_{-1}\left(x_{1}\right)}{dx^{\left(2\right)}}=\overset{1}{\underset{j=-2}{\sum }}w_{1,j}^{\left(1\right)}Q_{-1}^{\prime }\left(x_{j}\right),\\ \frac{d^{\left(2\right)}Q_{N+1}\left(x_{1}\right)}{dx^{\left(2\right)}}=\overset{N+3}{\underset{j=N}{\sum }}w_{1,j}^{\left(1\right)}Q_{N+1}^{\prime }\left(x_{j}\right),\\ \frac{d^{\left(3\right)}Q_{N+1}\left(x_{1}\right)}{\partial x^{\left(3\right)}}=\overset{N+3}{\underset{j=N}{\sum }}w_{1,j}^{\left(1\right)}Q_{N+1}^{\prime \prime }\left(x_{j}\right). \end{array}$$

By using these equations which we obtained by derivations, three unknown terms will be eliminated from the system. Consider

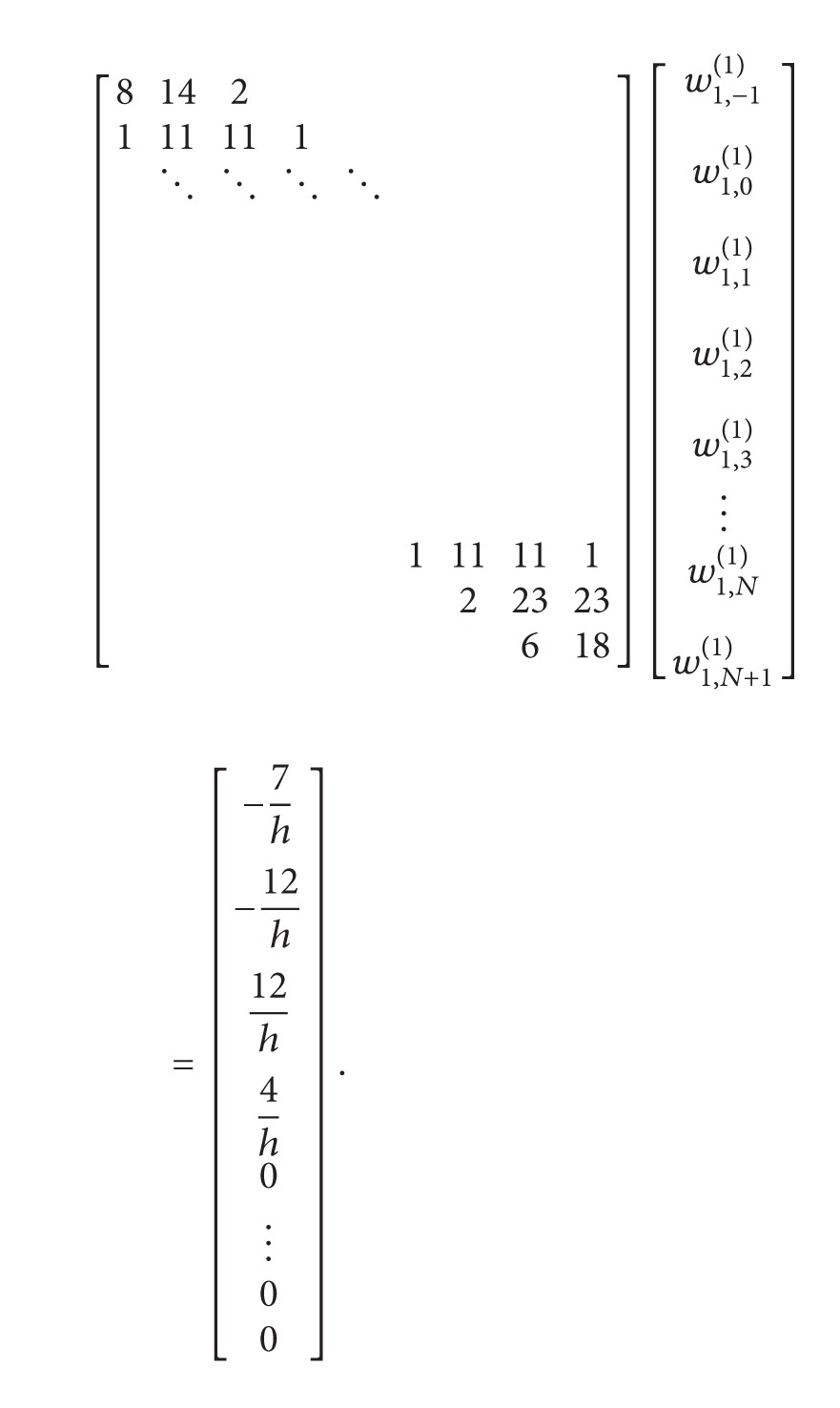
(33)


To determine the weighting coefficients, *w*_*k*,*j*_^(1)^, *j* = −1,0,…, *N* + 1, at grid points *x*_*k*_, 2 ≤ *k* ≤ *N* − 1, we got the following algebraic equation system:

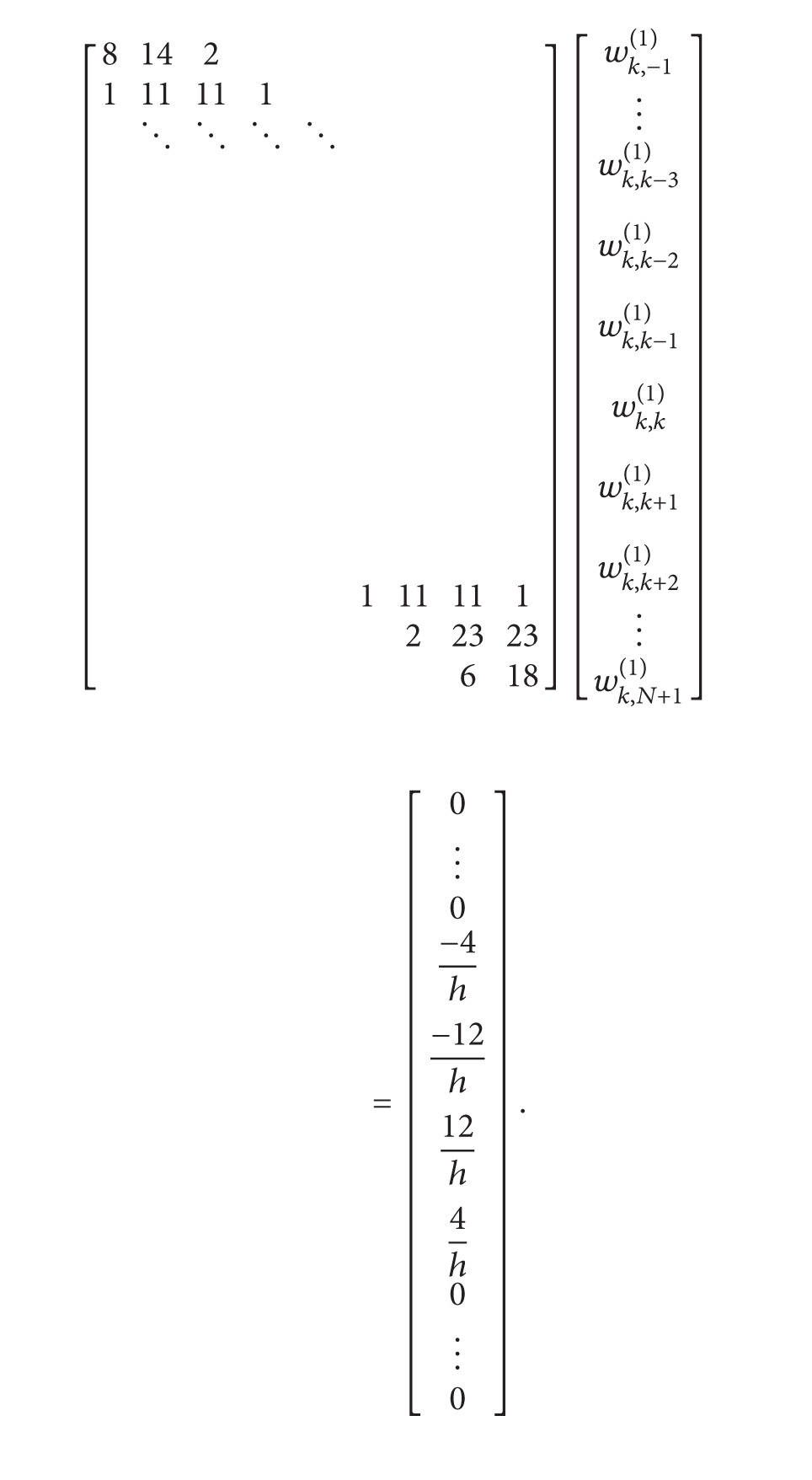
(34)


For the last grid point of the domain *x*_*N*_, determine weighting coefficients, *w*_*N*,*j*_^(1)^, *j* = −1,0,…, *N* + 1, we got the following algebraic equation system:

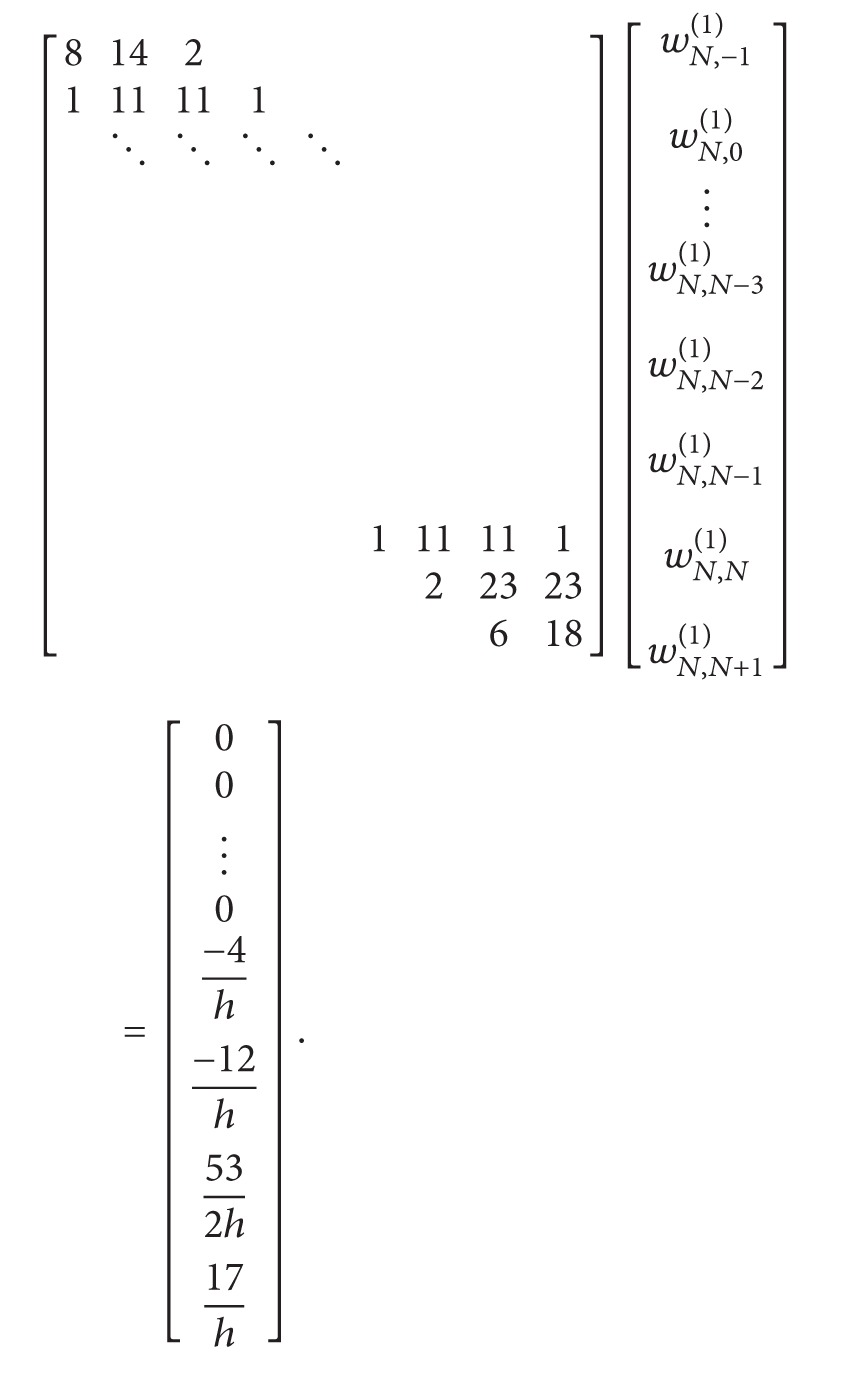
(35)


### 2.5. Second-Order Derivative Approximation

The general form of DQM approximation to the problem on the solution domain is

(36) d2Qmdx2(xi)=∑j=m−1m+2wi,j(2)Qm(xj),m=−1,0,…,N+1, i=1,2,…,N,

where *w*_*i*,*j*_^(2)^ represents the corresponding weighting coefficients of the second-order derivative approximations. Similarly, for finding out the weighting coefficients of the first grid point *x*_1_ all test functions *Q*_*m*_, *m* = −1,0,…, *N* + 1, are used and the following algebraic equations system is obtained:

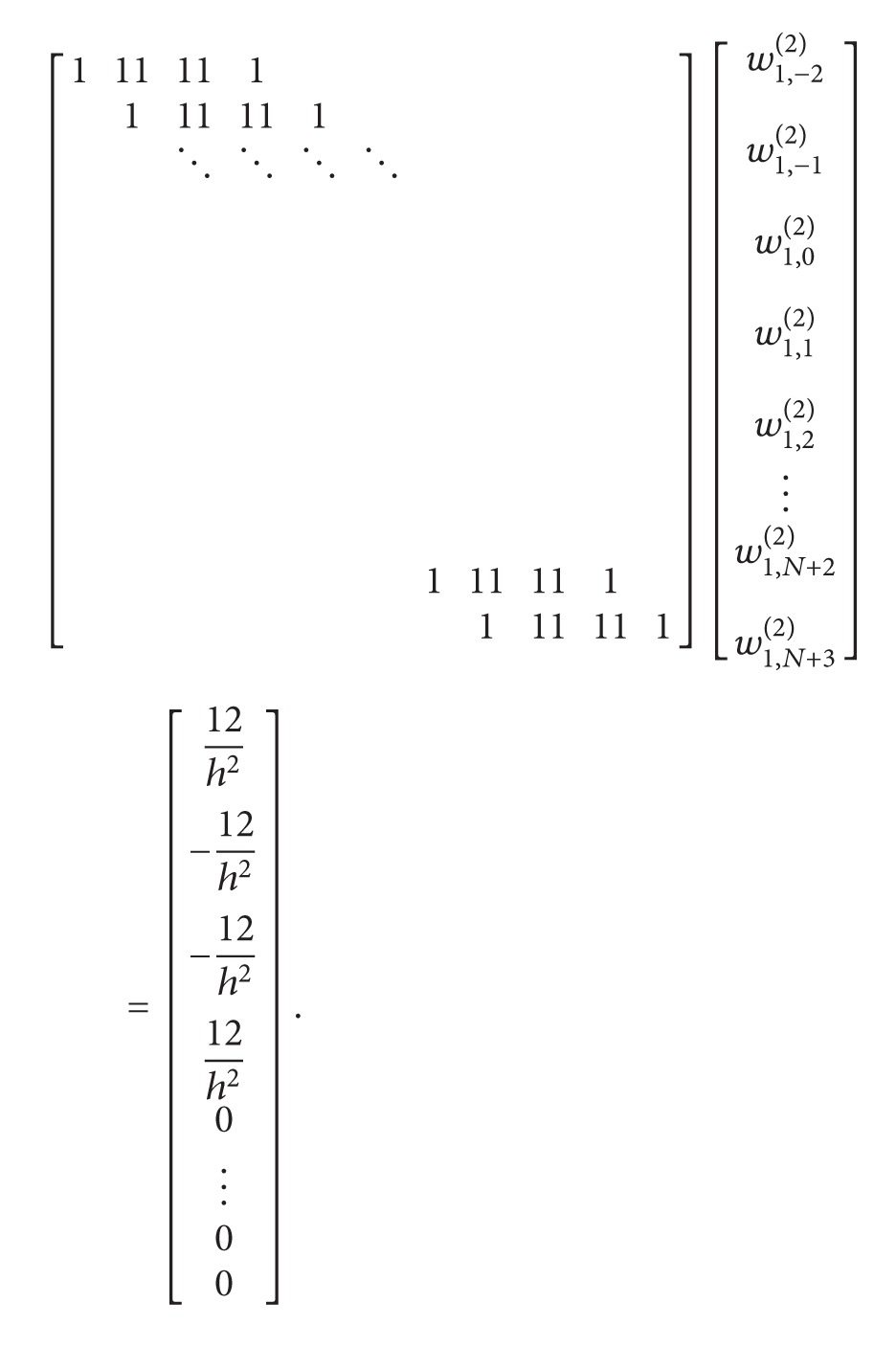
(37)


Here, the system (37) consists of *N* + 6 unknowns and *N* + 3 equations. To have a unique solution, it is required to add new equations to the system. These are

(38) $$\begin{array}{c} \frac{d^{3}Q_{-1}\left(x_{1}\right)}{dx^{3}}=\overset{1}{\underset{j=-2}{\sum }}w_{1,j}^{\left(1\right)}Q_{-1}^{\prime }\left(x_{j}\right), \end{array}$$

(39) $$\begin{array}{c} \frac{d^{3}Q_{N+1}\left(x_{1}\right)}{dx^{3}}=\overset{N+3}{\underset{j=N}{\sum }}w_{1,j}^{\left(1\right)}Q_{N+1}^{\prime }\left(x_{j}\right). \end{array}$$

If we used (38) and (39) we obtain the following equations system:

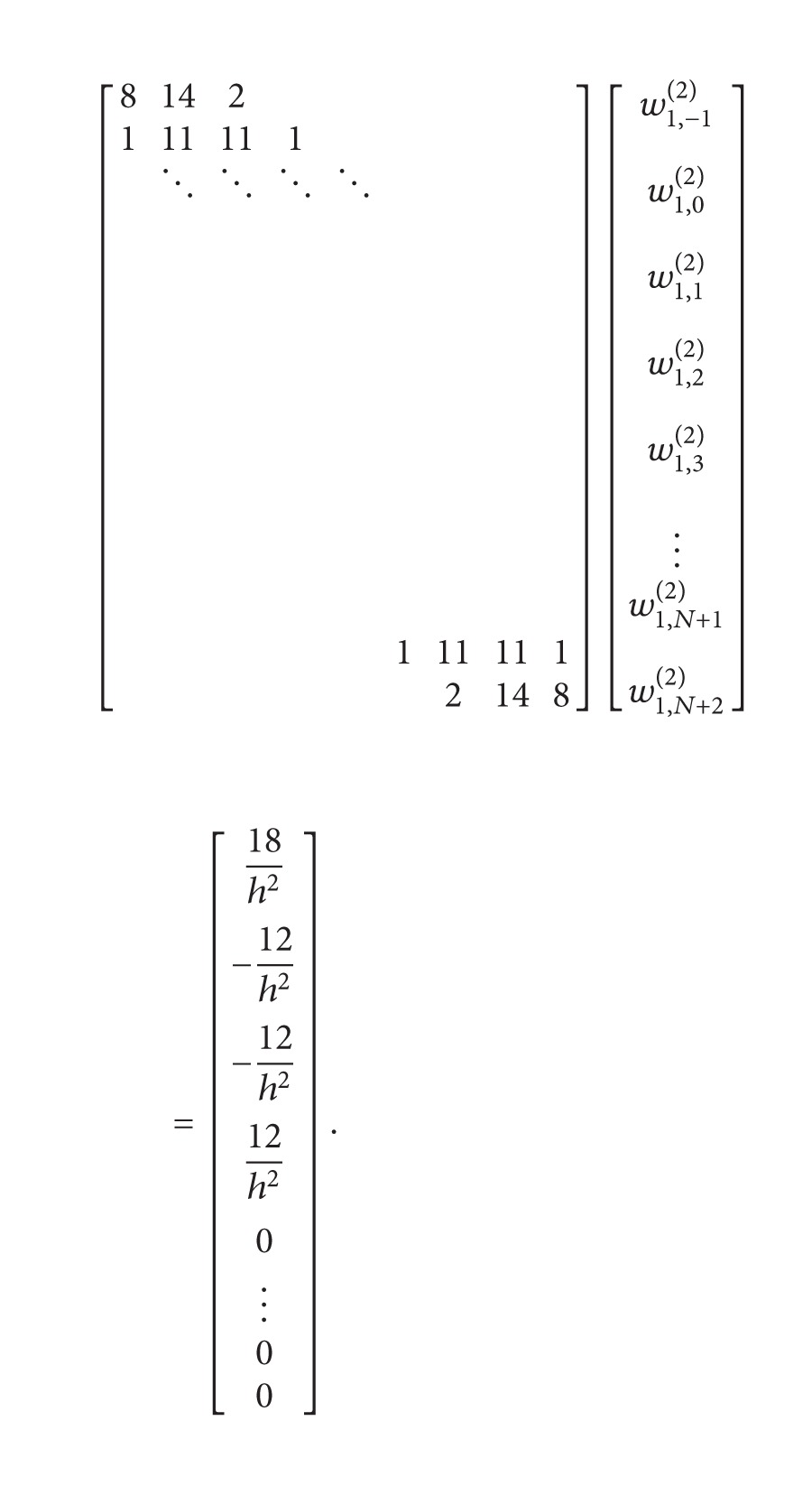
(40)
Quartic B-splines have not got fourth-order derivations at the grid points so we cannot eliminate the unknown term *w*_1,*N*+2_^(2)^ by the one more derivation of (39). We will use first-order weighting coefficients instead of second-order weighting coefficients which are introduced by Shu [32]

(41) $$\begin{array}{c} w_{i,j}^{\left(2\right)}=2w_{i,j}^{\left(1\right)}\left(w_{i,i}^{\left(1\right)}-\frac{1}{x_{i}-x_{j}}\right),\;i\neq j. \end{array}$$

After we use (41),

(42) A1=w1,N+2(2)=2w1,N+2(1)(w1,1(1)−1x1−xN+2),[8142111111⋱⋱⋱⋱11111214][w1,−1(2)w1,0(2)w1,1(2)w1,2(2)w1,3(2)⋮w1,N(2)w1,N+1(2)]  =[18h2−12h2−12h212h20⋮−A1−8A1]

system (42) is obtained. To determine the weighting coefficients *w*_*k*,*j*_^(2)^, *j* = −1,0,…, *N* + 1, at grid points *x*_*k*_, 2 ≤ *k* ≤ *N* − 1, we got the following algebraic system:

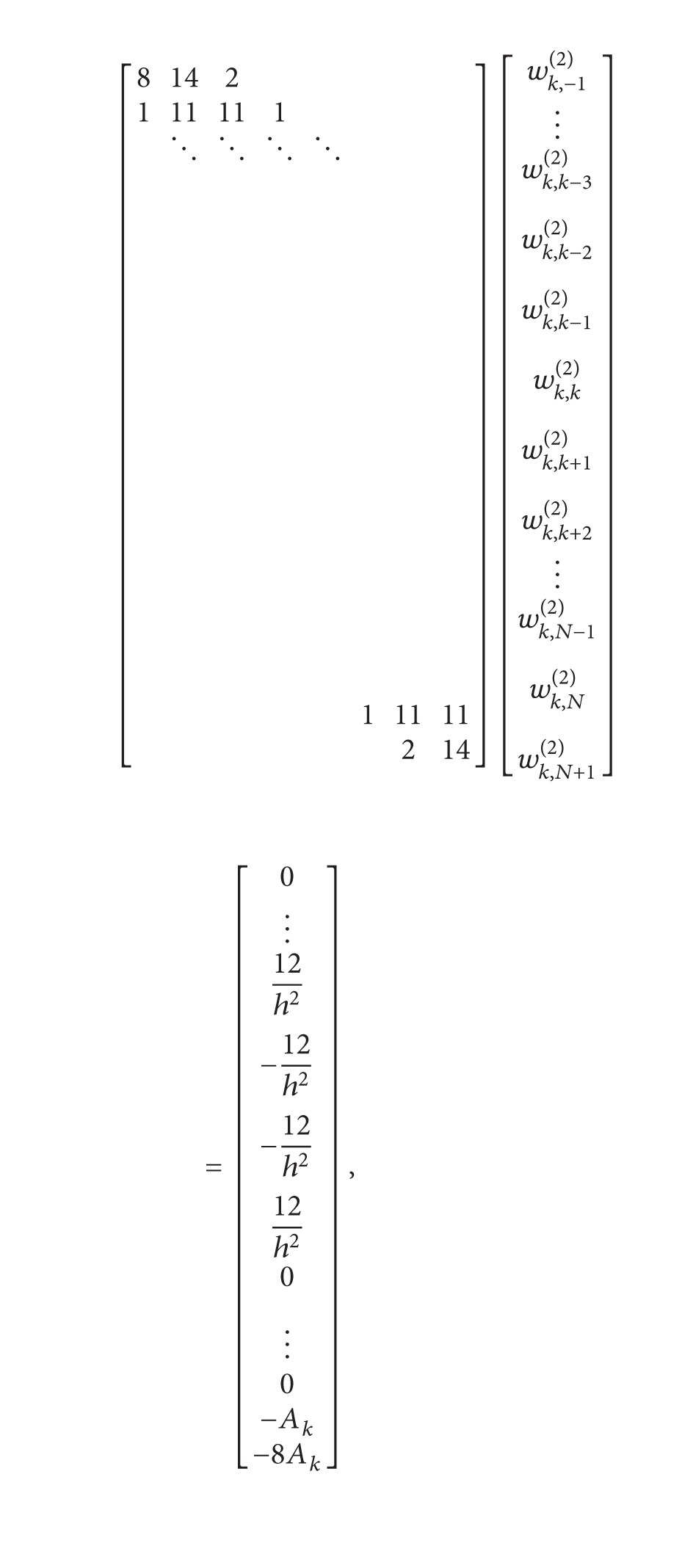
(43)
where *A*_*k*_ equals *A*_*k*_ = *w*_*k*,*N*+2_^(2)^ = 2*w*_*k*,*N*+2_^(1)^(*w*_*k*,*k*_^(1)^ − 1/(*x*_*k*_ − *x*_*N*+2_)).

For the last grid point of the domain *x*_*N*_ with the same idea, determine weighting coefficients *w*_*N*,*j*_^(2)^, *j* = −1,0,…, *N* + 1, we got the following algebraic equation system:

(44) [8142111111⋱⋱⋱⋱11111214][wN,−1(2)wN,0(2)⋮wN,N−3(2)wN,N−2(2)wN,N−1(2)wN,N(2)wN,N+1(2)]  =[00⋮012h2−12h2−12h2−AN18h2−8AN],

where *A*_*N*_ equals *A*_*N*_ = *w*_*N*,*N*+2_^(2)^ = 2*w*_*N*,*N*+2_^(1)^(*w*_*N*,*N*_^(1)^ − 1/(*x*_*N*_ − *x*_*N*+2_)).

## 3. Test Problem and Experimental Results

In this section, we obtained numerical solutions of the MBE by the subdomain finite element method and differential quadrature method. The accuracy of the numerical method is checked using the error norms *L*_2_ and *L*_*∞*_, respectively,

(45) L2=||Uexact−UN||2≃h∑J=1N|Ujexact−(UN)j|2,L∞=||Uexact−UN||∞≃max⁡j|Ujexact−(UN)j|,j=1,2,…,N−1.

All numerical calculations were computed in double precision arithmetic on a Pentium 4 PC using a Fortran compiler. The analytical solution of modified Burgers' equation is given in [33] as

(46) $$\begin{array}{c} U\left(x,t\right)=\frac{\left(x/t\right)}{1+\left(\sqrt{t}/c_{0}\right)\exp\left(x^{2}/4vt\right)}, \end{array}$$

where *c*_0_ is a constant and 0 < *c*_0_ < 1. For our numerical calculation, we take *c*_0_ = 0.5. We use the initial condition for (46), evaluating at *t* = 1, and the boundary conditions are taken as *U*(0, *t*) = *U*_*x*_(0, *t*) = 0 and *U*(1, *t*) = *U*_*x*_(1, *t*) = 0.

### 3.1. Experimental Results for FEM

**Figure 1:**
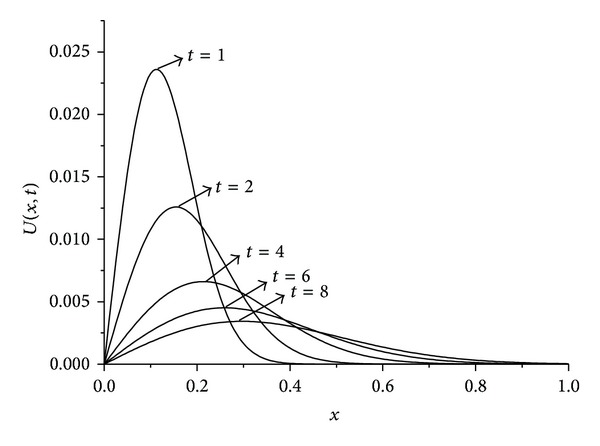
Solution behavior of the equation with *h* = 0.005, *t* = 0.01, and *v* = 0.01.

**Figure 2:**
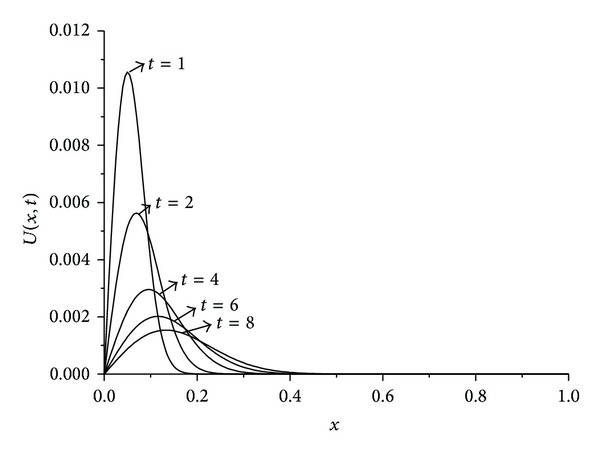
Solution behavior of the equation with *h* = 0,005, *t* = 0,01, and *v* = 0.001.

**Figure 3:**
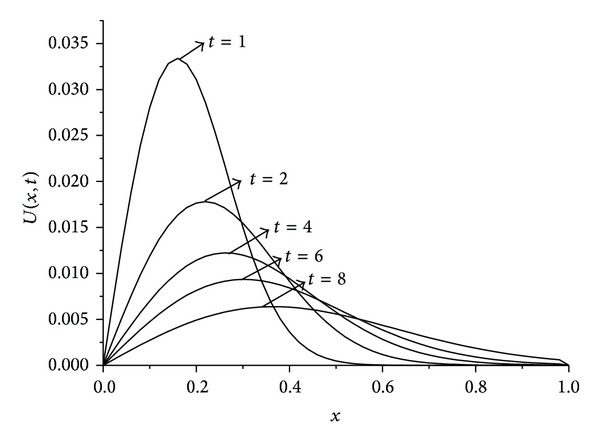
Solution behavior of the equation with *h* = 0,02, *t* = 0,01, and *v* = 0.01.

For the numerical simulation, we have chosen the various viscosity parameters *v* = 0.01,0.001,0.005, space steps *h* = 0.02,0.005, and time steps Δ*t* = 0.01,0.001 over the interval 0 ≤ *x* ≤ 1. The computed values of the error norms *L*_2_ and *L*_*∞*_ are presented at some selected times up to *t* = 10. The obtained results are tabulated in Tables 1, 2, 3, and 4. It is clearly seen that the results obtained by the SFEM are found to be more acceptable. Also, we observe from these tables that if the value of viscosity decreases, the value of the error norms will decrease. When the value of viscosity parameter is smaller we get better results. The behaviors of the numerical solutions for viscosity *v* = 0.01, 0.005, 0.001, space steps *h* = 0.02, 0.005, and time steps Δ*t* = 0.01, 0.001 at times *t* = 1,2, 4,6, and 8 are shown in Figures 1, 2, and 3. As seen in the figures, the top curve is at *t* = 1 and the bottom curve is at *t* = 8. Also, we have observed from the figures that as the time increases the curve of the numerical solution decays. With smaller viscosity value, numerical solution decay gets faster.

### 3.2. Experimental Results for QBDQM

We calculate the numerical rates of convergence (ROC) with the help of the following formula:

(47) $$\begin{array}{c} \text{ROC}\approx \frac{\ln\left(E\left(N_{2}\right)/E\left(N_{1}\right)\right)}{\ln\left(N_{1}/N_{2}\right)}. \end{array}$$

Here *E*(*N*_*j*_) denotes either the *L*_2_ error norm or the *L*_*∞*_ error norm in case of using *N*_*j*_ grid points. Therefore, some further numerical runs for different numbers of space steps have been performed. Ultimately, some computations have been made about the ROC by assuming that these methods are algebraically convergent in space. Namely, we suppose that *E*(*N*) ~ *N* ^*p*^ for some *p* < 0 when *E*(*N*) denotes the *L*_2_ or the *L*_*∞*_ error norms by using *N* subintervals.

**Figure 4:**
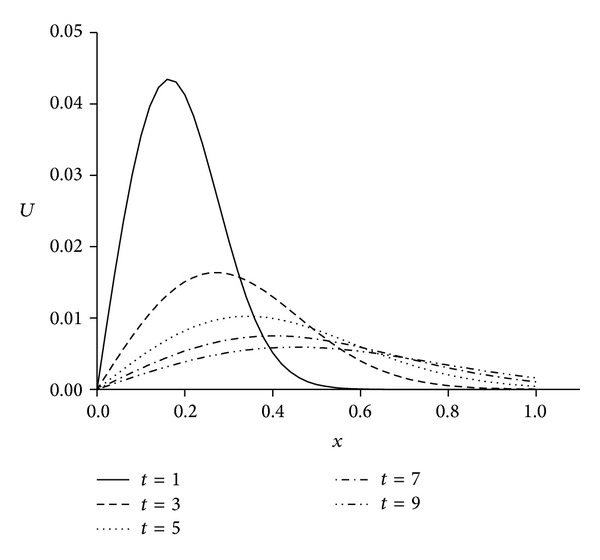
Solutions for *v* = 0.01, *h* = 0.02, Δ*t* = 0.01, and 0 ≤ *x* ≤ 1.

**Figure 5:**
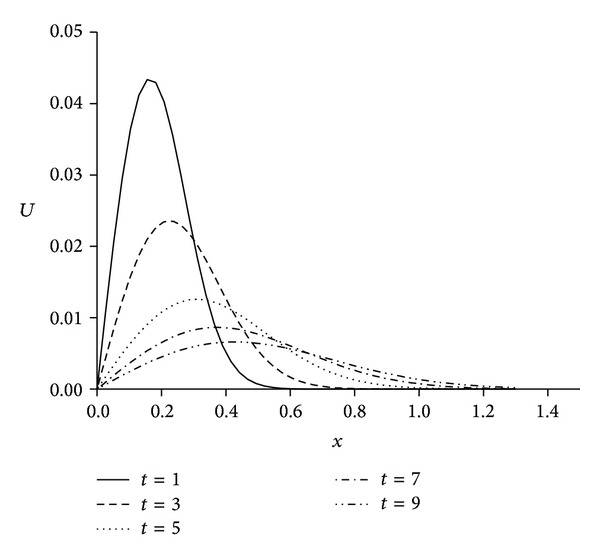
Solutions for *v* = 0.01, *h* = 0.02, Δ*t* = 0.01, and 0 ≤ *x* ≤ 1.3.

**Figure 6:**
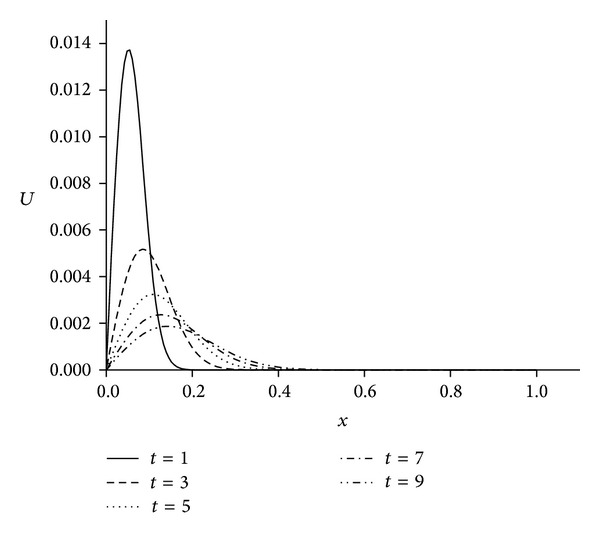
Solutions for *v* = 0.001, Δ*t* = 0.01, *N* = 166, and 0 ≤ *x* ≤ 1.

**Figure 7:**
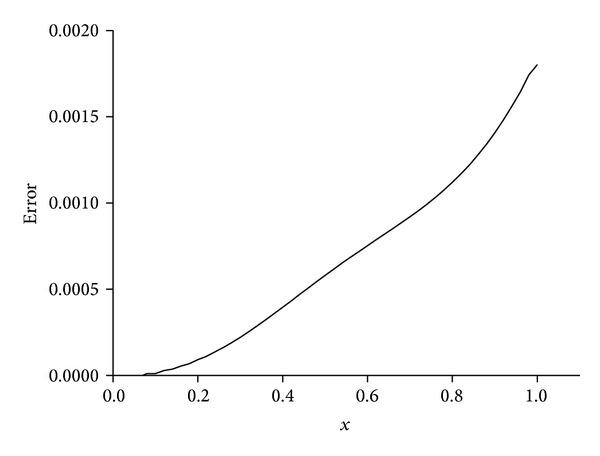
Errors for *v* = 0.01, Δ*t* = 0.01, *h* = 0.02, and 0 ≤ *x* ≤ 1.

**Figure 8:**
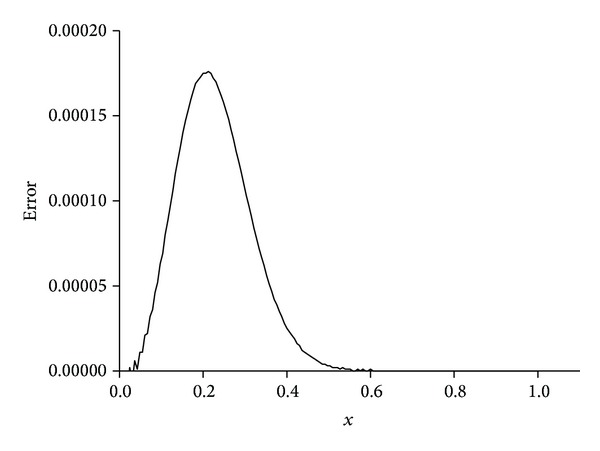
Errors for *v* = 0.001, Δ*t* = 0.01, and *N* = 166, 0 ≤ *x* ≤ 1.

For the numerical treatment, we have taken the different viscosity parameters *v* = 0.01,0.001 and time step Δ*t* = 0.01 over the intervals 0 ≤ *x* ≤ 1 and 0 ≤ *x* ≤ 1.3. As it is seen from Figure 4 when we select the solution domain 0 ≤ *x* ≤ 1 the right part of the shock wave cannot be seen clearly. By using the larger domain like 0 ≤ *x* ≤ 1.3 as seen in Figure 5 solution is got better than narrow domain 0 ≤ *x* ≤ 1 shown in Figure 4. The computed values of the error norms *L*_2_ and *L*_*∞*_ are presented at some selected times up to *t* = 10. The obtained results are recorded in Tables 5 and 6. As it is seen from the tables, the error norms *L*_2_ and *L*_*∞*_ are sufficiently small and satisfactorily acceptable. We obtain better results if the value of viscosity parameter is smaller, as shown in Table 7. The behaviors of the numerical solutions for viscosity *v* = 0.01 and 0.001 and time step Δ*t* = 0.01 at times *t* = 1,3, 5,7, and 9 are shown in Figures 4–6. It is observed from the figures that the top curve is at *t* = 1 and the bottom curve is at *t* = 9. It is obviously seen that smaller viscosity value *v* in shock wave with smaller amplitude and propagation front becomes smoother. Also, we have seen from the figures that, as the time increases, the curve of the numerical solution decays. With smaller viscosity value, numerical solution decay gets faster. These numerical solutions graphs also agree with published earlier work [13]. Distributions of the error at time *t* = 10 are drawn for solitary waves, Figures 7 and 8, from which maximum error happens at the right hand boundary when greater value of viscosity *v* = 0.01 is used, and with smaller value of viscosity *v* = 0.001, maximum error is recorded around the location where shock wave has the highest amplitude. The *L*_2_ and *L*_*∞*_ error norms and numerical rate of convergence analysis for *v* = 0.001 and Δ*t* = 0.01 and different numbers of grid points are tabulated in Table 8.

Table 9 presents a comparison of the values of the error norms obtained by the present methods with those obtained by other methods [13, 14, 17, 18]. It is clearly seen from the table that the error norm *L*_2_ obtained by the SFEM is smaller than those given in [13, 14, 17, 18] whereas the error norm *L*_*∞*_ is very close to those given in [14, 17, 18]. The error norm *L*_*∞*_ is better than the paper [13]. For the QBDQM both *L*_2_ and *L*_*∞*_ are almost the same as these papers.

## 4. Conclusion

In this paper, SFEM and DQM based on quartic B-splines have been set up to find the numerical solution of the MBE (2). The performance of the schemes has been considered by studying the propagation of a single solitary wave. The efficiency and accuracy of the methods were shown by calculating the error norms *L*_2_ and *L*_*∞*_. Stability analysis of the approximation obtained by the schemes shows that the methods are unconditionally stable. An obvious conclusion can be drawn from the numerical results that for the SFEM *L*_2_ error norm is found to be better than the methods cited in [13, 14, 17, 18] whereas *L*_*∞*_ error norm is found to be very close to values given in [13, 14, 17, 18]. The obtained results show that our methods can be used to produce reasonable accurate numerical solutions of modified Burgers' equation. So these methods are reliable for getting the numerical solutions of the physically important nonlinear problems.

## Figures and Tables

**Table 1:** *L*_2_ and *L*_∞_ error norms for *h* = 0.005, Δ*t* = 0.01, and *v* = 0.001 (SFEM).

Time	*L*_2_ × 10^3^	*L*_*∞*_ × 10^3^
2	0.0054945	0.0282049
3	0.0082404	0.0422421
4	0.0109858	0.0562280
5	0.0137296	0.0701566
6	0.0164729	0.0840427
7	0.0192154	0.0978975
8	0.0219573	0.1116934
9	0.0246985	0.1254466
10	0.0274379	0.1391304

**Table 2:** *L*_2_ and *L*_∞_ error norms for *h* = 0.005, Δ*t* = 0.001, and *v* = 0.005 (SFEM).

Time	*L*_2_ × 10^3^	*L*_∞_ × 10^3^
2	0.0246966	0.0845689
3	0.0370384	0.1266222
4	0.0493707	0.1684362
5	0.0616997	0.2101319
6	0.0740253	0.2516392
7	0.0863444	0.2930178
8	0.0986573	0.3341922
9	0.1109636	0.3752457
10	0.1232629	0.4160477

**Table 3:** *L*_2_ and *L*_∞_ error norms for *h* = 0.005, Δ*t* = 0.01, and *v* = 0.01 (SFEM).

Time	*L*_2_ × 10^3^	*L*_∞_ × 10^3^
2	0.0978574	0.2806243
3	0.1467089	0.4185981
4	0.1955072	0.5550286
5	0.2442506	0.6898713
6	0.2929396	0.8238629
7	0.3415703	0.9566688
8	0.3901436	1.0881289
9	0.4386580	1.2182231
10	0.4871136	1.3469237

**Table 4:** *L*_2_ and *L*_∞_ error norms for *h* = 0.02, Δ*t* = 0.01, and *v* = 0.01 (SFEM).

Time	*L*_2_ × 10^3^	*L*_∞_ × 10^3^
2	0.0973818	0.2802526
3	0.1460008	0.4184872
4	0.1945704	0.5554121
5	0.2430873	0.6910062
6	0.2915506	0.8252312
7	0.3399602	0.9580433
8	0.3883156	1.0894413
9	0.4366131	1.2194111
10	0.4848547	1.3479880

**Table 5:** *L*_2_ and *L*_∞_ error norms for *v* = 0.01, Δ*t* = 0.01, and *h* = 0.02.

Time	QBDQM [*h* = 0.02]		Ramadan et al. [13] [*h* = 0.02]	
	*L*_2_ × 10^3^	*L*_∞_ × 10^3^	*L*_2_ × 10^3^	*L*_∞_ × 10^3^
2	0.7955855586	1.3795978925	0.7904296620	1.7030921188
3	0.6690533313	1.1943543646	0.6551928290	1.1832698216
4	0.5250528343	0.9764154381	0.5576794264	0.9964523368
5	0.4048512821	0.7849457015	0.5105617536	0.8561342445
6	0.3452210304	0.6374950443	0.5167229575	0.7610530060
7	0.3638648688	0.6705419608	0.5677438614	1.0654548090
8	0.4337013450	0.9863405006	0.6427542266	1.3581113635
9	0.5197862999	1.2551335234	0.7236430257	1.6048306653
10	0.6042925888	1.4747885309	0.8002564201	1.8023938553

**Table 6:** *L*_2_ and *L*_∞_ error norms for *v* = 0.01, Δ*t* = 0.01, and *N* = 81 at 0 ≤ *x* ≤ 1.3.

Time	QBDQM	
	*L*_2_ × 10^3^	*L*_∞_ × 10^3^
2	0.7607107169	1.3704182195
3	0.6480181273	1.1854984190
4	0.5604986926	1.0052476452
5	0.4927784148	0.8654032419
6	0.4359075842	0.7531551023
7	0.3885737191	0.6601326512
8	0.3520185942	0.5833334970
9	0.3282544303	0.5201323663
10	0.3187570280	0.4691560472

**Table 7:** *L*_2_ and *L*_∞_ error norms for *v* = 0.001, Δ*t* = 0.01, and *h* = 0.005.

Time	QBDQM		Ramadan et al. [13]	
	*L*_2_ × 10^3^	*L*_∞_ × 10^3^	*L*_2_ × 10^3^	*L*_∞_ × 10^3^
2	0.1370706949	0.4453892504	0.1835491190	0.8185211112
3	0.1168507335	0.3842839811	0.1441424335	0.5234833346
4	0.1019761971	0.3258391192	0.1144110783	0.3563537207
5	0.0920706001	0.2816616769	0.0947865272	0.2549790058
6	0.0849484881	0.2484289381	0.0814174677	0.2134847835
7	0.0794570772	0.2225471690	0.0718977757	0.1880048432
8	0.0750035859	0.2019577762	0.0648368942	0.1682601770
9	0.0712618898	0.1851510002	0.0594114970	0.1524074966
10	0.0680382860	0.1711033543	0.0551151456	0.1394312127

**Table 8:** Error norms and rate of convergence for various numbers of grid points at *t* = 10.

*N*	*L*_2_ × 10^3^	ROC(*L*_2_)	*L*_∞_ × 10^3^	ROC(*L*_∞_)
11	0.43	—	0.98	—
21	0.35	0.31	0.88	0.16
31	0.22	1.19	0.52	1.35
41	0.17	0.92	0.39	1.02
51	0.14	0.88	0.30	1.20
81	0.10	0.72	0.19	0.98

**Table 9:** Comparison of our results with earlier studies.

Values and methods	*L*_2_ × 10^3^	*L*_∞_ × 10^3^	*L*_2_ × 10^3^	*L*_∞_ × 10^3^
	*t* = 2	*t* = 2	*t* = 10	*t* = 10
*v* = 0.005, Δ*t* = 0.001, *h* = 0.005				
SFEM	0.02469	0.08456	0.12326	0.41604
[14]	0.25786	0.72264	0.18735	0.30006
[17] SBCM1	0.22890	0.58623	0.14042	0.23019
[17] SBCM2	0.23397	0.58424	0.13747	0.22626
*v* = 0.001, Δ*t* = 0.01, *h* = 0.005				
SFEM	0.00549	0.02820	0.02743	0.13913
QBDQM	0.13707	0.44538	0.06803	0.17110
[13]	0.18354	0.81852	0.05511	0.13943
[14]	0.06703	0.27967	0.05010	0.12129
[17] SBCM1	0.06843	0.26233	0.04080	0.10295
[17] SBCM2	0.07220	0.25975	0.03871	0.09882
[18]	0.06607	0.26186	0.04160	0.10470
*v* = 0.01, Δ*t* = 0.01, and *h* = 0.005				
SFEM	0.09785	0.28062	0.48711	1.34692
[14]	0.52308	1.21698	0.64007	1.28124
[17] SBCM1	0.38489	0.82934	0.54826	1.28127
[17] SBCM2	0.39078	0.82734	0.54612	1.28127
[18]	0.37552	0.81766	0.19391	0.23074
*v* = 0.01, Δ*t* = 0.01, and *h* = 0.02				
SFEM	0.09738	0.28025	0.48485	1.34798
QBDQM	0.79558	1.37959	0.60429	1.47478
[13]	0.79042	1.70309	0.80025	1.80239
[17] SBCM1	0.38474	0.82611	0.55985	1.28127
[17] SBCM2	0.41321	0.81502	0.55095	1.28127
